# Stepwise Reduction of Graphene Oxide (GO) and Its Effects on Chemical and Colloidal Properties

**DOI:** 10.1038/s41598-018-28353-6

**Published:** 2018-07-04

**Authors:** Samar Azizighannad, Somenath Mitra

**Affiliations:** 1 Department of Materials Science and Engineering, New Jersey institute of Technology, Newark, New Jersey 07102 USA; 2 Department of Chemistry and Environmental Sciences, New Jersey institute of Technology, Newark, New Jersey 07102 USA

## Abstract

Graphene Oxides (GO) typically contains different oxygen containing groups such as hydroxyl, carboxyl and epoxy, and reduced GO (r-GO) represents a family of material with diverse chemical properties. In an effort to understand how properties of r-GO change as GO is reduced, a stepwise reduction of the same GO to r-GO containing different levels of oxygen was carried out, and their corresponding chemical and colloidal properties are reported. Starting with GO containing 49 percent oxygen, r-GOs containing 31, 19 and 9 percent oxygen were synthesized. The aqueous behavior in terms of solubility gradually decreased from 7.4 µg/ml for GO to nearly zero for r-GO with 9% oxygen, while dispersibility under sonication decreased from 8 to 2.5 µg/ml for the same samples. Hydrophobicity index as measured as the octanol water partition coefficient decreased from −3.89 to 5.2% as oxygen content dropped from 49 to 9%. Colloidal behavior was also dramatically affected by reduction, and critical coagulation concentration (CCC) dropped from 28 to 15 in presence of 0.5 mmole/l NaCl and from 6 to 2 in presence of 0.5 mmole/l MgCl_2_ as the oxygen in the original GO was reduced to 9%.

## Introduction

Graphene-based materials have unique optical, mechanical and electrical properties which make them attractive for many applications^1–5^. Oxidation of graphite powder to graphene oxide (GO) followed by chemical reduction to reduced graphene oxide (r-GO) is a well-established approach to generating graphene based materials. Numerous methods of chemical reduction to r-GO have been published where hydrazine hydrate^6^, dimethylhydrazine^3^, hydroquinone^3^, NaBH_4_^7^, HI^3,7^ and Fe and Zn powder^3,8^ have been used to reduce GO. Since the GO from different sources vary widely and contains a wide range of oxygen containing groups such as hydroxyl, carboxyl and epoxy^2–29^, r-GO represents a family of material with different physical/chemical properties. While there are several reports on different aspects and applications of GO and r-GO^8,27,28,30,31^ where GO and r-GO were used from diverse sources, a systematic study of physical and chemical property variation in r-GO containing different levels of oxygen from the same GO is yet to be studied.

The level of reduction in r-GO is also expected to alter aqueous dispersibility of these species. Besides chemical behavior, this also has ecological consequences. As the applications of GO and r-GO proliferate, mass production and disposal of products containing these nanocarbon will increase with potential for environmental contamination and water pollution. Recent studies have shown that graphene can be toxic toward organisms including bacteria^32^, nematodes zebra fish and humans^32,33^. Cytotoxicity toward bacteria through both membrane and oxidative stress has been demonstrated for both GO and r-GO and level of oxidation has been shown affect cytotoxicity^32,34^. While a hydrophobic r-GO can be expected to settle out of aqueous media into solid phases such as river sediments, hydrophilic r-GO will stay dispersed. Therefore, there is a need to develop an understanding of the fate of different r-GOs in aqueous media. There have been very limited reports on this topic^10,22,23,29^, and the variation in colloidal behavior with oxygen content in r-GO is yet to be studied. Similarly, aqueous behavior in terms of solubility, dispersibility and hydrophobicity of these r-GOs is not well understood.

Theoretical predictions of GO aggregation kinetics and stability using Derjaguin− Landau− Verwey− Overbeek (DLVO) theory has been used to estimate the attachment efficiency^9,10,14^, and an alternative Maxwell approach^35^ taking into account has also been used to determine particle collision efficiencies and aggression kinetics of GO and r-GOs. Time resolved dynamic light scattering has been used to study dispersibility of GO and r-GOs^36^, and the solubility of r-GO from different methods have been measured in different solvents and correlated with solubility parameters^5^.

An important consideration is that oxygen containing groups play an important role in determining chemical properties as well as dispersibility and aggregation of r-GO in aqueous solutions. Since GO and r-GO form different sources show a wide range of variability in both structure as well as the presence of functional groups, they cannot be compared directly. To address this issue, the objective of this work is the stepwise reduction of the same GO to generate r-GO containing different levels of oxygen and study their chemical properties. The use of the same GO to form r-GO eliminates the variability associated with different sources of this highly diverse material. Yet another objective is to study the colloidal behavior of the r-GOs representing different levels of GO reduction.

## Results and Discussions

**Figure 1:**
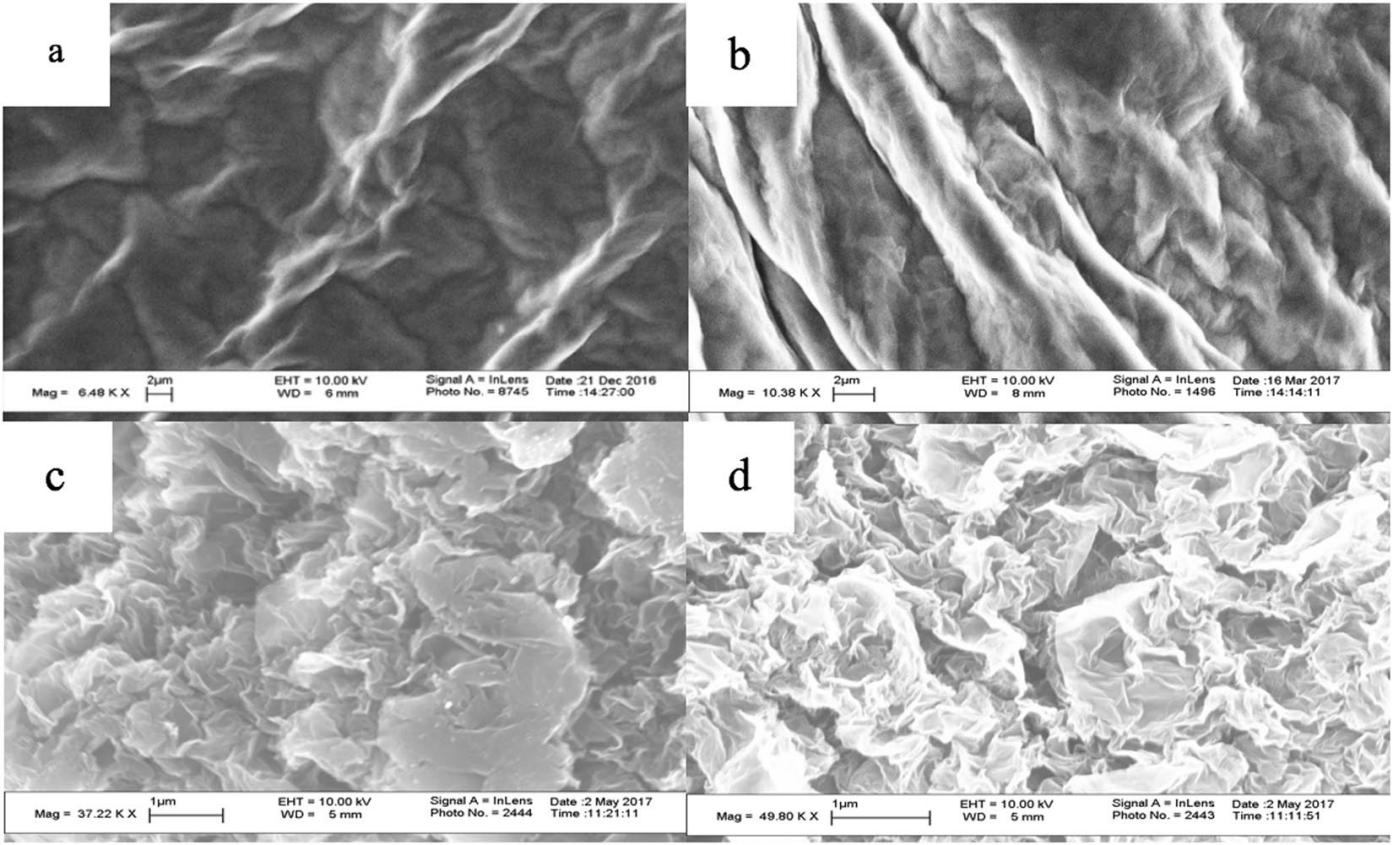
SEM images of (**a**) GO, (**b**) r-GO-31, (**c**) r-GO-19, (**d**) r-GO-9.

To avoid the inter sample variation of GO from different sources, the same sample was reduced step wise using a gentle reduction technique. r-GOs containing 31, 19 and 9% oxygen were synthesized. The resulting r-GOs are listed in Table 1 and these were classified based on the oxygen content. The oxygen content was measured using Elemental Analysis(EA), Thermo Gravimetric Analysis(TGA) and energy-dispersive X-ray(EDAX). There were minor variations among these measurements, and EA was accepted to be the true value. The scanning electron microscopy(SEM) images of the different r-GO are presented in Fig. 1. In line with what has been reported before, the GO sheets had smooth surface while the r-GOs showed folded regimes and wrinkles^37^.

**Table 1:** Properties of GO and r-GOs produced via stepwise reduction.

Analysis/Sample	GO	r-GO-31	r-GO-19	r-GO-9
Percent Carbon	47.48%	66.87%	80.06%	87.71%
Percent Oxygen	49%	31.67%	19.11%	9.69%
L_a_	22.6	18.5	16	13.4
Particle size in 0.5 mmole/1 NaCl(nm)	642.3	385.5	376.7	327.9
CCC in NaCl	28	27	20	15
Particle size in 0.5 mmole/1 MgCl_2_(nm)	1274	608.2	551.1	358.3
CCC in MgCl_2_	6	6	5	2
Zeta potential in 0.5 mmole/1 NaCl	−33.2	−30.02	−29.5	−23.7
Zeta potential in 0.5 mmole/1 MgCl_2_	−9.66	−4.54	−2.2	−0.92
Hydrophobicity Index	−3.89%	0.98%	1.75%	5.2%
Solubility(µg/ml)	7.4	2.1	~0	~0
Dispersibility(µg/ml)	8	6.3	4.1	2.5

**Figure 2:**
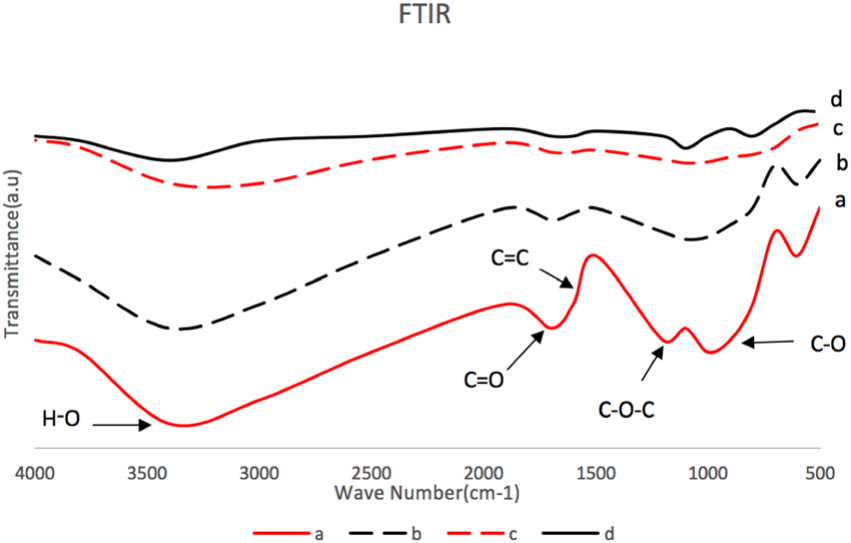
FTIR spectra of (**a**) GO, (**b**) r-GO-31, (**c**) r-GO-19, (**d**) r-GO-9.

The chemical structure of GO before and after the reduction were studied by Fourier Transform Infrared spectroscopy (FTIR) and the data is presented in Fig. 2. The reduction of GO involves the elimination of oxygen containing groups and the restoration of conjugated π systems. Characteristic peaks including C–O (1060 cm^−1^), C–OH (1226 cm^−1^), O–H (1412 cm^−1^) and C = O (1733 cm^−1^), were observed in the GO spectrum, and these were significantly reduced in the r-GO spectra. This clearly indicated the loss of oxygen groups form GO suggesting the formation of r-GO.

**Figure 3:**
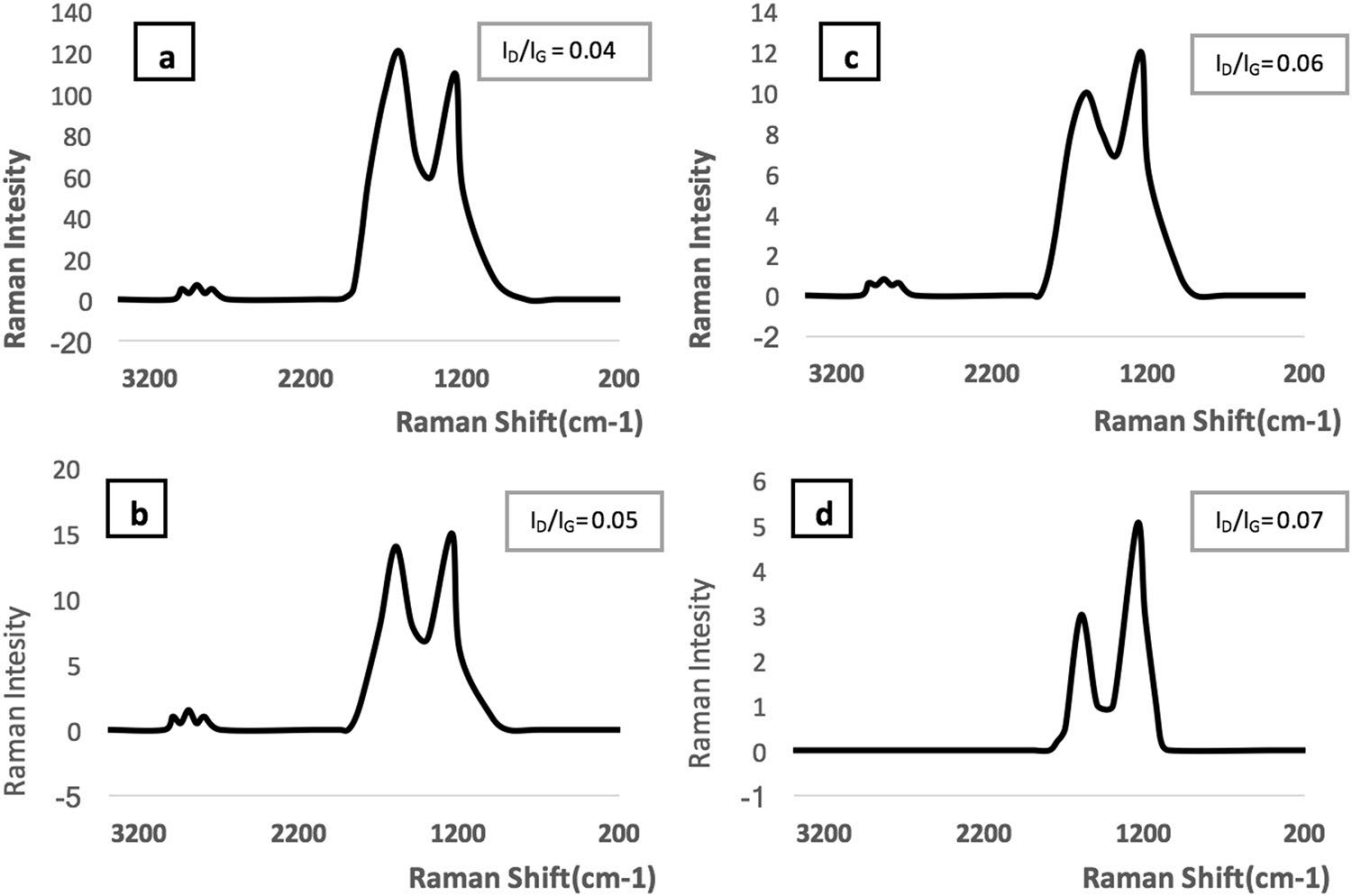
Raman spectra of (**a**) GO, (**b**) r-GO-31, (**c**) r-GO-19, (**d**) r-GO-9 (The I_D_/I_G_ ratio as abstract value for in-plane lattice defects).

Figure 3 shows the Raman spectra of the GO and r-GO samples. Strong D-peak is suggestive of arm chair conformation near the edges. After the reduction of GO, the G band shifted to 1580 cm^−1^ from 1600 cm^−1^ in line with what has been reported before^8^. The intensity of the D band increased with reduction and so did the I_D_/I_G_ ratio. The crystallite sizes (L_a_) of the sp^2^ lattice of all the samples were calculated from the Eq. 1, where λ is the laser wavelength, and I_G_ and I_D_ are the intensities of the G- band and the D- band respectively.

1 $${L}_{a}=2.4\times {10}^{-10}\times {\lambda }_{laser}^{4}\times {I}_{G}/{I}_{D}$$

The intensity of D band increased during reduction while the intensity of G band decreased. As result L_a_ decreased in r-GO. The values of L_a_ are given in Table 1, which shows that L_a_ decreased from 22.6 in original GO to 13.4 in r-GO-9.

**Figure 4:**
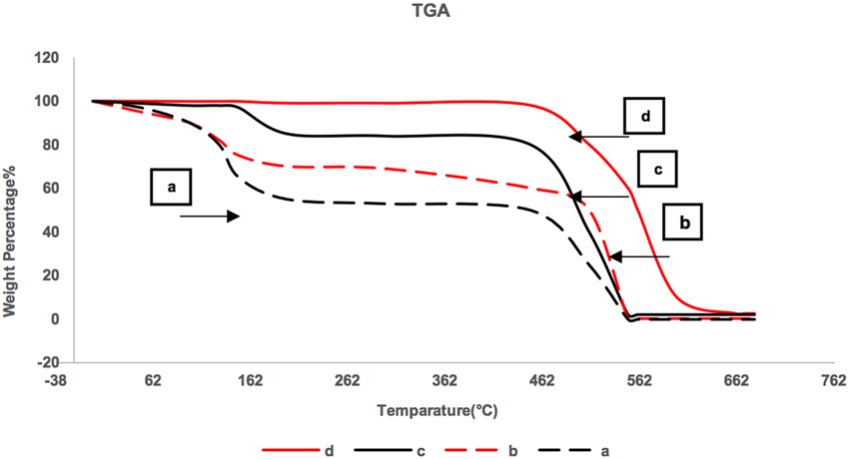
TGA curves of (**a**) GO, (**b**) r-GO-31, (**c**) r-GO-19, (**d**) r-GO-9.

The results from TGA analysis are presented in Fig. 4. The two weight loss steps for GO were from the pyrolysis of oxygen containing functional groups and the second was from the oxidation of carbon. The former was around 160 °C and GO lost nearly 40% of its weight at 162 °C. The second step which is around 460 °C was related to the oxidation of sp^2^-hybridzied carbon atoms. As the reduction progressed, the weight loss at 160 °C decreased with r-GO-9 showing minimal decrease in weight at this temperature indicating that much of the oxygen containing groups had been removed. As reported previously^38^, deoxygenation also led to higher thermal stability of the r-GO. As the oxygen concentration decreased to 9%, the residual oxygen containing groups were more stable and oxidized at a higher temperature and more slowly. Thus, r-GO-9 showed the highest thermal stability among the ones presented here.

To measure solubility and dispersibility of GO and r-GOs, pre-weighed amounts of GO and r-GOs were added to DI water and let the solution settle for 2 hours. As shown in Table 1 solubility of graphene oxide reduces from 7.4 µg/ml to nearly zero for r-GO-9. However, the r-GO could be dispersed into a stable suspension via sonication. Dispersibility was measured by sonicating the suspension for 10 min and then the suspension was allowed to settle for 24 hours. As presented in in Table 1, here the dispersibility decreased from 8 µg/ml for GO to 2.5 µg/ml for r-GO-9.

**Figure 5:**
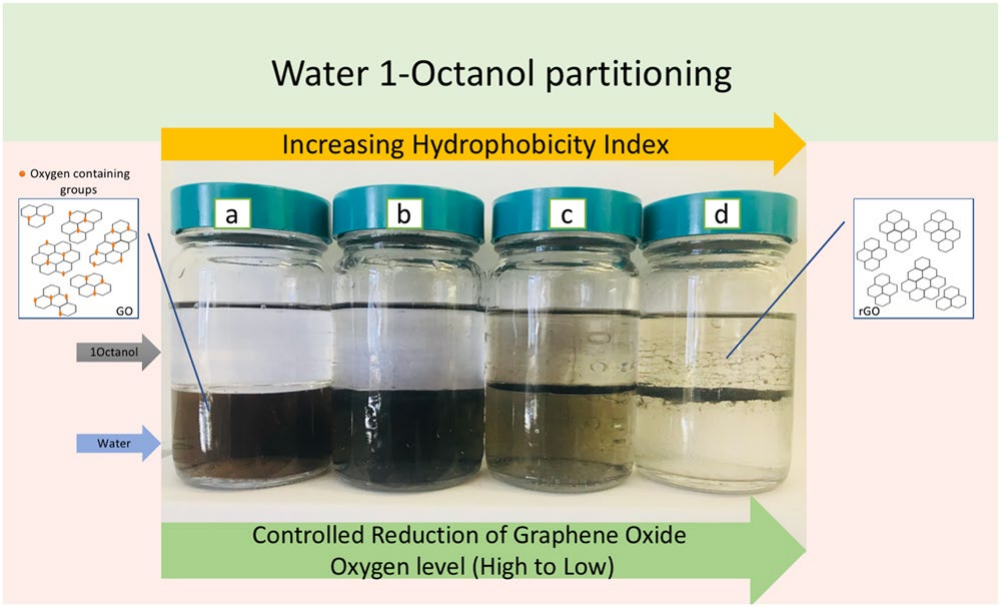
Photographs of 1-octanol/water partitioning of (**a**) GO, (**b**) r-GO-31, (**c**) r-GO-19, (**d**) r-GO-9 after standing for an hour.

In the realm of aqueous behavior, the hydrophobicity of the different r-GOs is an important consideration. Hydrophobicity Index(HI) based on octanol water partitioning was used to determine dispersibility of GO in water^39^. The pictures of 1-octanol/water partitioning are shown in Fig. 5. HI was calculated based using a method published before^36^. It was computed using absorbance of GO and r-GOs solutions at 252 nm in water prior to and following 1-octanol extraction according the formula 2^39^.

2 $$HI\,( \% )=\frac{({A}_{0}-{A}_{i})}{{A}_{0}}\times 100$$

As presented in Table 1, as oxygen content in r-GOs decreased, hydrophobicity increased. Hydrophilicity was high in highly carboxylated GO sheets which made it partition in the aqueous phase. HI increased from −3.89% to 5.2% as the oxygen content decreased implying that the it went from a highly hydrophilic GO to highly hydrophobic r-GO which represented a dramatic change in aqueous dispersibility. HI of GO (−3.89%) is very close to reported HI for reported carboxylated carbon nanotubes (−4.15%)^39^.

**Figure 6:**
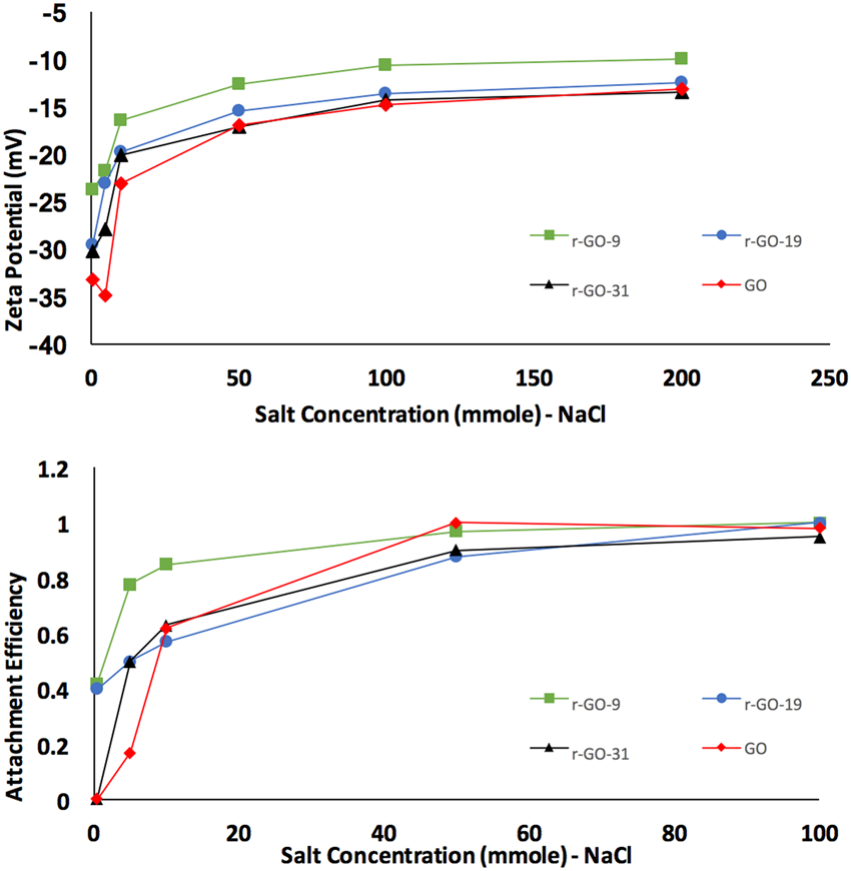
(**a**) Zeta potential as a function of NaCl concentration, (**b**) Attachment efficiency as a function of NaCl concentration. GO and r-GOs concentration was maintained 4 mg/1 in DI water.

**Figure 7:**
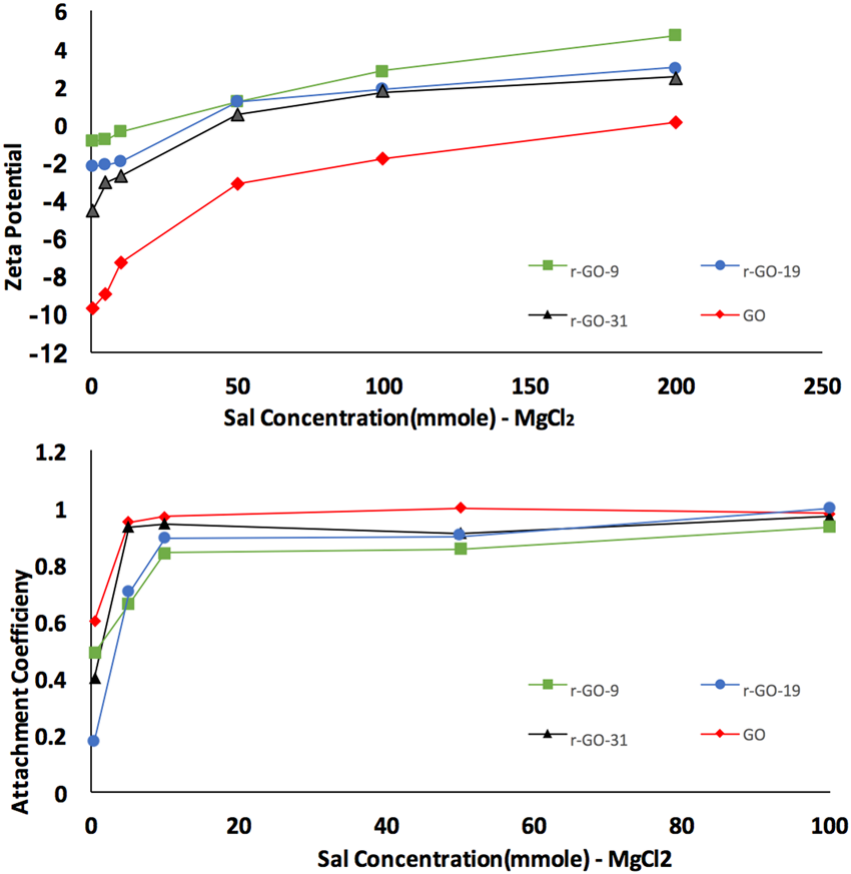
(**a**) Zeta potential as a function of MgCl2 concentration; (**b**) Attachment efficiency as a function of MgCl2 concentration. GO and r-GOs concentration were maintained 4 mg/1 in DI water.

In general, colloidal stability is attributed to balance between van der Waals forces that promote aggregation and electrostatic repulsion which is dispersive^36^. Zeta potential, particle size distribution of agglomerates and aggregation kinetics were used to study dispersibility of the different r-GO (Table 1). Due to its anisotropic shape, the two fundamental interacting modes between GO sheets are edge to edge and face to face. GO is known to form a good dispersion in water because of the electrostatic repulsion between ionized functionalities such as carboxylic groups that are mainly located at the edges. With the addition of salt, typically the monovalent Na^+^ has no specific interactions with the functional groups on GO surfaces and the aggregation follows conventional DLVO theory. In the presence of divalent Mg^2+^, the mechanisms of GO aggregation kinetics could be complicated because the divalent cations can also interact with surface functional groups of the GO sheets and even cross-link them, particularly at the edges^14,36,39^. Figures 6, 7 show zeta potential and particle size of GO and r-GOs as a function of ionic strength. As expected, the GO and r-GO particles began to aggregate with increase in ionic strength. The addition of a divalent cation Mg^2+^ led to stronger aggregation of the GO sheets which is in line with the DLVO theory. As presented in Table 1, agglomerate size in the presence of 0.5 mmole of NaCl and MgCl_2_ increased as oxygen content increased. Particle size reduces from 642.3 to 327.9 nm in presence of 0.5 mmole/1 NaCl and from 1274 to 358.3 nm in presence of equivalent MgCl_2_ as oxygen containing groups are being removed. The zeta potential in NaCl was between 33.2 to 23.7 mV, which implied moderately stable dispersions, however the zeta potential in 0.5 mmole/1 MgCl_2_ was in the range of 9.66 to 0.92 mV, implying very unstable suspension.

The aggregation kinetics of the GO and r-GO were studied using time resolved dynamic light scattering. In Eq. 3, the initial rate of agglomerate size is (r_h_) is proportional to kn_o_ where k is the initial aggregation rate constant and n_o_ is the initial concentration of the solute. The attachment efficiency α which is the reciprocal of stability ratio of a dispersion were computed NaCl and MgCl_2_ as^36^:

3 $$\propto =\frac{{(\frac{drh}{dt})}_{t\to 0}}{{(\frac{dr\,h}{dt})}_{t\to 0}^{(f)}}$$

where ${(\frac{drh}{dt})}_{t\to 0}$ and ${(\frac{dr\,h}{dt})}_{t\to 0}^{(f)}$ represent the slow and fast aggregation regimes respectively^36^. The attachment efficiency was measured as the ratio of the initial slope of the aggregation profile to that obtained under fast aggregation conditions. These are plotted as a function of salt concentration for the GO and r-GO and are presented in Figs 6 and 7. In line with previous studies, distinct unfavorable and favorable aggregation regimes demarcated by the critical coagulation concentration (CCC) (Figs 6, 7) were observed. This indicated that the DLVO type interactions were the dominant mechanism for colloidal stability of GO and r-GOs. The CCC values are presented in Table 1. Surface oxidation in r-GO clearly played an important role and higher oxygen content led to higher CCC values. There was no significant change in CCC value for GO and r-GO-31. Higher CCC for high oxygen containing groups was due to the fact that there was more interaction between oxygen containing group and electrolytes in solution. In general, the CCC value determined for GO and r-GO is significantly lower than the reported CCC value for fullerene but is quiet similar to CNT^32^.

## Methods

### Sample Preparation

Graphene Oxide was purchased from Graphena Inc., Zinc was purchased from Fluka and all other chemicals were purchased from Sigma Aldrich with purity higher than 95%. Reduction of GO to r-GO was carried out using a method published before^8^, however the method was modified for step-wise reduction by adding different amount of Zn to the solution. 200 mg of GO was dispersed in 50 ml water and sonicated for 10 min to form a homogeneous solution. 0.1 M Hydrochloric acid was added to adjust the pH to 2. The Zn power was then added and sonicated for 10 min to generate enough hydrogen that would lead to formation of r-GO. Reducing the amount of Zn reduced the hydrogen generation and consequently the degree of reduction the addition of 200, 400 and 1000 mg Zn led to the formation r-Go containing 31, 19 and 9 percent oxygen respectively. These are referred to as r-GO-31, r-GO-19 and r-GO-9. The reduction of GO took place according to the Eqs 4 and 5. Any remaining Zn was dissolved by adding additional HCl before filtration:

4 $$Z{\rm{n}}+2{\rm{HCl}}\to {{\rm{ZnCl}}}_{2}+{{\rm{H}}}_{2}$$

5 $${\rm{GO}}+{{\rm{H}}}_{2}\to {\rm{rGO}}+{{\rm{H}}}_{2}{\rm{O}}$$

### Characterization of The Reduced Graphene Oxide

The GO and r-GOs were analyzed using scanning electron microscope (SEM), energy-dispersive X-ray (EDAX), Elemental Analysis (EA), Raman Spectroscopy, Thermo Gravimetric Analysis (TGA), and Fourier Transform Infrared spectroscopy (FTIR). SEM analysis was carried out on a LEO 1530 VP instrument equipped with an energy-dispersive X-ray; TGA was performed on Pyris 1 system from Perkin-Elmer Corp., EA analysis was carried out by Perkin-Elmer 2400 Series II analyzer, and FTIR measurements were carried out in purified KBr pellets using a Perkin-Elmer (Spectrum One) instrument. TGA analysis was carryout by heating from 30 °C to 700 °C under a flow of air at 10 mL/min, at a heating rate of 2 °C per min.

Stock solutions of GO and r-GOs were prepared by sonication. Pre-weighed amounts of the GO and r-GOs were added to MilliQ water to make a 40 mg/1 stock solution. Different GO and r-GO solutions were then prepared by diluting the stock solution. Stock solutions containing 400 mM of sodium chloride and magnesium chloride were also prepared which were used for dispersibility studies. Dynamic Light Scattering (DLS) were carried out using 50 mg/1 dispersions of r-GO were measured as a function of salt concentration at 25°c using dynamic light scattering (Malvern Instruments Zetasizer Nano ZS90). The dynamic light scattering measurements were conducted at 90° with the incident laser beam and the autocorrelation function having been allowed to accumulate for more than 10 s with salt concentration ranging between 0.5 mM and 200 mM. Zeta potential of the Graphene oxide was measured on 10 mg l-1 dispersions at 25° on the Malvern Instriments Zetasizer nano ZS90. Hydrophobicity of GO and r-GOs were determined by measuring the UV absorbance at 252 nm before and after partitioning in water extraction of a 50 mg l-1 dispersion of the GO with 1-octanol.

## Conclusions

Controlled, step wise reduction of GO was carried out by nascent hydrogen generated from a reaction between metallic zinc and HCl. r-GOs containing 31, 19 and 9% oxygen were synthesized and studied. FTIR confirmed the reduction of GO while Raman and SEM showed increase in defects and wrinkles in r-GOs. Aqueous dispersibility and colloidal behavior as measured by size of agglomerates, zeta potential r-GO were highly dependent on oxygen content. Higher oxygen content led to higher CCC values in both NaCl and MgCl_2_.
